# Loss of imprinting of *IGF2* correlates with hypermethylation of the *H19* differentially methylated region in hepatoblastoma

**DOI:** 10.1038/sj.bjc.6604754

**Published:** 2008-10-28

**Authors:** S Honda, Y Arai, M Haruta, F Sasaki, M Ohira, H Yamaoka, H Horie, A Nakagawara, E Hiyama, S Todo, Y Kaneko

**Affiliations:** 1 Department of Cancer Diagnosis, Saitama Cancer Center, Research Institute for Clinical Oncology, 818 Komuro, Ina, Saitama 362-0806, Japan; 2 Cancer Genomics Project, National Cancer Center Research Institute, Chuo-Ku, Tokyo 104-0045, Japan; 3 Japanese Study Group for Pediatric Liver Tumor (JPLT), Hiroshima 734-8551, Japan; 4 Department of General Surgery, Hokkaido University Graduate School of Medicine, Sapporo 060-8638, Japan

**Keywords:** hepatoblastoma, *IGF2*, *H19*, loss of heterozygosity, loss of imprinting

## Abstract

*IGF2*, a maternally imprinted foetal growth factor gene, is implicated in many childhood tumours including hepatoblastoma (HB); however, the genetic and epigenetic alterations have not comprehensively been studied. We analysed the methylation status of the *H19* differentially methylated region (DMR), loss of heterozygosity (LOH) and allelic expression of *IGF2* in 54 *HB* tumours, and found that 12 tumours (22%) with LOH, 9 (17%) with loss of imprinting (LOI) and 33 (61%) with retention of imprinting (ROI). Biallelic and monoallelic *IGF2* expressions correlated with hypermethylation and normal methylation of *H19* DMR, respectively, in two tumours with LOI and seven tumours with ROI. Quantitative RT–PCR analysis showed minimal expression of *H19* mRNA and substantial expression of *IGF2* mRNA in tumours with LOH or LOI, and substantial expression of both *H19* and *IGF2* mRNAs in tumours with ROI. Increased *IGF2* expression with predominant embryonic P3 transcript was found in the majority of HBs with ROI and foetal livers. In contrast to the earlier reports, our findings suggest that the disruption of the enhancer competition model reported in Wilms' tumour may also occur in HB. Both frequencies of LOH and LOI seem to be lower in HB than in Wilms' tumour, reflecting the different tissue origins.

Hepatoblastoma (HB) is a rare malignant neoplasm of the liver, with an incidence of 0.5–1.5 per million children (Perilongo and Shafford, 1999). Remarkable progress in clinical outcome has been achieved in the past 20 years because of advances in chemotherapy and surgical procedures; however, the mortality rate remains 20–30% and treatment results in patients in advanced stages who are refractory to standard preoperative chemotherapy regimens are unsatisfactory (Perilongo *et al*, 2000; Fuchs *et al*, 2002). To improve the mortality of these patients, innovative treatment based on a specific molecular target is needed. The molecular mechanism involved in the development and progression of HB includes overexpression of insulin-like growth factor-II (*IGF2*) (Li *et al*, 1998b; Gray *et al*, 2000; Hartmann *et al*, 2000), downregulation of *RASSF1A* by promoter hypermethylation (Sugawara *et al*, 2007; Honda *et al*, 2008) and alterations of genes in the Wnt signalling pathway; most notably, the high incidence of *CTNNB1* (catenin, *β*1) mutation (Koch *et al*, 1999; Taniguchi *et al*, 2002).

*IGF2* is a maternally imprinted gene and encodes a foetal peptide hormone that regulates cellular proliferation and differentiation (Foulstone *et al*, 2005). *IGF2* has four promoter regions and P3 is the most active promoter in the foetal liver, followed by P2 and P4 promoters (Li *et al*, 1998a). *PLAG1* encodes a developmentally regulated transcription factor, which positively regulates *IGF2* through binding the P3 promoter region. Although *IGF2* is downregulated in normal tissues after birth, except for liver tissues, it is overexpressed in a wide variety of childhood and adult cancers and serves as a tumour enhancer through autocrine and paracrine mechanisms (Toretsky and Helman, 1996). *IGF2* has been studied extensively over the past decade as a key molecule involving HB and Wilms' tumour (WT) pathogenesis.

The allelic expression of *IGF2* is regulated by the methylation status of the sixth CTCF (CCCTC-binding factor) site in the *H19* differentially methylated region (DMR) that represents the parental origin of the *IGF2* allele; whereas the paternal CTCF6 allele is methylated, the maternal allele is unmethylated in normal tissues (Bell and Felsenfeld, 2000; Hark *et al*, 2000; Takai *et al*, 2001). Using the enhancer competition model, *IGF2* and *H19* promoters compete on the same chromosome for a shared enhancer, and access of the maternal *IGF2* allele to this enhancer is blocked by *H19* DMR when unmethylated because of the insulator activity of CTCF binding to unmethylated *H19* DMR (Bell and Felsenfeld, 2000; Hark *et al*, 2000). It has been proved in many WTs that aberrant methylation of the maternal CTCF6 prevents the insulator binding and leads to loss of imprinting (LOI), resulting in the overexpression of *IGF2* (Steenman *et al*, 1994; Ravenel *et al*, 2001). Although LOI of *IGF2* was reported in HB, the mechanism of LOI, the concurrent overexpression of *IGF2* mRNA and loss of *H19* mRNA expression are uncertain because of the limited number of HB tumours examined and the low frequency of the heterozygous *IGF2* polymorphic site in general populations (Davies, 1993; Montagna *et al*, 1994; Rainier *et al*, 1995; Li *et al*, 1995, 1998b; Fukuzawa *et al*, 1999; Gray *et al*, 2000; Hartmann *et al*, 2000; Ross *et al*, 2000; Albrecht *et al*, 2004; Suzuki *et al*, 2008), and some investigators stated earlier that the mechanisms of *IGF2* upregulation by LOI found in WT do not apply to HB (Li *et al*, 1995; Hartmann *et al*, 2000).

Loss of imprinting was reported in 32–38% of WTs (Ravenel *et al*, 2001; Fukuzawa *et al*, 2004; Yuan *et al*, 2005), and loss of heterozygosity (LOH), leading to uniparental disomy (UPD) of the paternal *IGF2*, was reported in 36–50% of WTs (Grundy *et al*, 1996; Fukuzawa *et al*, 2004; Yuan *et al*, 2005). In HB, although LOH of *IGF2* was reported in 20–30%, the incidence of LOI of *IGF2* was uncertain because each series included only a small number of HB tumours. In addition, it is also uncertain whether the same mechanism of LOI is involved in both WT and HB tumorigeneses because the methylation status of *H19* DMR in HB has rarely been examined (Li *et al*, 1995, 1998b; Fukuzawa *et al*, 1999).

To determine whether the alterations of *IGF2* and *H19* loci identified in WT are also found in HB, we examined the LOI and LOH status of *IGF2* using combined bisulphite restriction assay (COBRA) of the CTCF6 region that can determine the methylation status of *H19* DMR more efficiently than the method using methylation-specific restriction enzymes and Southern blot in 54 HB tumours. In addition, we evaluated promoter-specific *IGF2* transcripts, the methylation status of *IGF2* promoters and *PLAG1* mRNA expression. Our results showed that the genetic and epigenetic alterations in the *IGF2-H19* region with elevated expression of *IGF2* mRNA identified in WTs were also found in the great majority of HB tumours, although the incidences of LOH and LOI may be lower in HBs than in WTs.

## Materials and methods

### Patients and samples

Tumour tissues were obtained from 54 Japanese children with HB, and adjacent normal liver tissues were available from 5 patients. Eighteen tumour and five matched normal liver specimens were supplied by the Tissue Bank of the Japanese Study Group for Pediatric Liver Tumour (JPLT) (Matsunaga *et al*, 2004), and 36 were supplied by institutions affiliated with Saitama Cancer Center. DNA and RNA were extracted from tumour and normal tissue samples that were immediately frozen after the resection or on arrival at the centre. The median age of the 54 patients at diagnosis was 18 months (range, 1–156 months). None of patients had the Beckwith–Wiedemann syndrome or a family history of familial polyposis coli. A total of 14 and 37 tumours were obtained before and after chemotherapy, respectively, and the chemotherapy status was unknown in the other 3 tumours. Pathologists in each institution and/or the JPLT pathology panel made the diagnosis of HB and verified that each sample contained 70% or more tumour cells. Informed consent was obtained from the parents, and the study design was approved by the ethics committee of Saitama Cancer Center.

### COBRA of the CTCF6 site at *H19* DMR

We performed COBRA to determine the methylation status of the CTCF6 binding site at *H19* DMR, as described earlier (Watanabe *et al*, 2007). COBRA of CTCF6 showed that the mean methylation percentage ±2 s.d. of five normal livers was 52.8±15.0%, and we defined more than the mean percentage +2 s.d. as the hypermethylated state.

### LOH analysis of *IGF2*

High-resolution single nucleotide polymorphism (SNP) array, Affymetrix Mapping 50K-Xba array (Affymetrix, Santa Clara, CA, USA), was used to analyse chromosomal aberrations of 11p15.5 where *IGF2* resides. Genomic DNA in 43 of 54 tumours and 2 cell lines was assayed according to the manufacturer's protocol, and the genomic status of *IGF2* was determined as described earlier (Haruta *et al*, 2008).

### Allelic expression analysis of *IGF2* and quantitative real-time reverse transcription-PCR analysis of *IGF2* and *H19* mRNA

The *Apa*I/*Ava*II polymorphic site in exon 9 of *IGF2* was used to evaluate the allelic expression of *IGF2* mRNA in 21 tumours whose RNA was available for this study, as described earlier (Watanabe *et al*, 2006). Quantitative real-time reverse transcription-PCR was performed to evaluate the total *IGF2* and *H19* mRNA levels in 20 tumour tissues, 2 HB cell lines (HuH6 and HepG2), foetal liver total RNA pooled from 34 foetuses (Clontech, Ohtsu, Japan) and 3 normal liver tissues adjacent to HB; the age of the patients was 16, 24 or 26 months. Of the 20 tumours, 3 and 16 were obtained before and after chemotherapy, respectively, and the chemotherapy status was unknown in 1. The primers and TaqMan probes used for *IGF2* and *H19* mRNA were described earlier (Watanabe *et al*, 2007; Haruta *et al*, 2008). The expression of *IGF2* and *H19* mRNAs was normalised with *GAPDH*.

### Methylation-specific PCR and bisulphite sequencing analysis of *IGF2* promoter regions

Genomic DNA from tumour and normal liver samples was treated with sodium bisulphite (Herman *et al*, 1996), and the methylation status of the P2–P4 promoter regions of *IGF2* was analysed by methylation-specific PCR (MSP), as described earlier (Beeghly *et al*, 2007). Polymerase chain reaction products were run on 2% agarose gels and visualised after staining with ethidium bromide. We confirmed the results of MSP analysis of P3 promoter by bisulphite sequencing of eight or more subcloned plasmids.

### Semiquantitative RT–PCR analysis of promoter-specific transcripts of *IGF2* and *PLAG1*

P1 and P3 promoter specific expressions of *IGF2* mRNA were analysed using the primer sets described elsewhere (Lu *et al*, 2006). The primer sequences for P2-specific transcript were derived from exons 4 and 5: forward, 5′-CCCTCAGGACGTGGACAG-3′; reverse, 5′-GTGCGTTGGACTTGCATAGA-3′; and the primer sequences for P4-specific transcript were derived from exons 7, 8 and 9: forward, 5′-CGAGCCTTCTGCTGAGCTAC-3′; reverse, 5′-CGGAAACAGCACTCCTCAAC-3′. *PLAG1* mRNA expression was analysed using the following primer sets: forward, 5′-AACGTAAGCGTGGTGAAACC-3′; reverse, 5′-TGCCACATTCTTCGCACTTA-3′ (Zatkova *et al*, 2004). Polymerase chain reaction products were run on polyacrylamide gels and visualised after ethidium bromide staining. The intensity of each band was examined using a fluorescence image analyser, FLA-3000G (Fujifilm, Tokyo, Japan). Dividing the intensity of the target transcript by that of *GAPDH* calculated the level of each transcript.

### Mutation analysis of the *CTNNB1* gene

To detect point mutations and deletions of the *CTNNB1* gene, genomic DNA from each tumour sample was amplified using two sets of primers, F1, 5′-TGGCTATCATTCTGCTTTTCTTG-3′ and R1, 5′-CTCTTTTCTTCACCACAACATTTT-3′, and BCAT-3, 5′-AAAATCCAGCGTGGACAATGG-3′ and BCAT-4, 5′-TGTGGCAAGTTCTGCATCATC-3′, respectively (Koch *et al*, 1999; Satoh *et al*, 2003). The PCR products were either directly sequenced or inserted into a vector (pGEM (R)-T Easy Vector System (Promega, Madison, WI, USA)), and six or more clones were sequenced.

### Statistical analysis

Student's *t*-test or Welch's *t*-test compared mRNA levels of *IGF2* and *H19* between tumours with or without *IGF2* alterations or other characteristics and the levels of *IGF2* promoter-specific transcripts between tumours with or without *PLAG1* mRNA expression. We also assessed the association between total *IGF2* mRNA levels and P2-, P3- or P4-specific *IGF2* mRNA levels by determining the Spearman rank correlation coefficient and associated *P*-value. Differences in the incidence of tumours with unmethylated P3 promoter were examined between tumours with hypermethylated *H19* DMR and tumours with normally methylated *H19* DMR by the *χ*^2^ test. Differences in the incidences of tumours with *CTNNB1* mutation were examined between any two of three groups of tumours classified on the basis of the *IGF2* status by the *χ*^2^ test.

## Results

### Methylation status of the CTCF6 binding site at *H19* DMR, LOH analysis using SNP array and allelic expression analysis of *IGF2*

**Figure 1:**
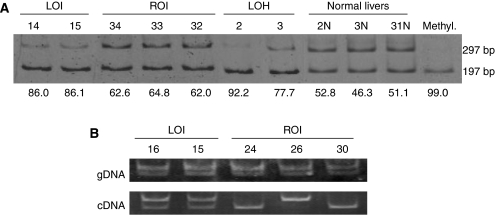
Analysis of *IGF2* alterations. (**A**) Examples of the methylation status of CTCF6 analysed by a combined bisulphite restriction assay (COBRA). Bisulphite-modified PCR products were digested with *Mlu*I. Upper and lower lanes indicate unmethylated and methylated fragments, respectively. Numbers above lanes indicate the tumour number. Numbers below lanes show the percentage of methylated DNA fragments containing CTCF6. The mean value of the DNA methylation percentages calculated from three COBRA experiments is shown in Table 1. Methyl., control methylated DNA. The *IGF2* status is shown above the tumour numbers. LOI, loss of *IGF2* imprinting; LOH, loss of heterozygosity in the *IGF2* region; ROI, retention of *IGF2* imprinting. (**B**) Electrophoretic pattern of genomic DNA PCR products or RT–PCR products after *Ava*II digestion. Reverse transcriptase–PCR analysis shows LOI in two tumours and ROI in three tumours.

Combined bisulphite restriction assay showed that 21 and 33 tumours had hypermethylation and normal methylation at CTCF6, indicating LOH or LOI and retention of *IGF2* imprinting (ROI), respectively (Table 1 and Figure 1). Single nucleotide polymorphism array analysis was performed in 43 of 54 tumours; all 21 tumours with hypermethylated CTCF6 and 22 of 33 tumours with normally methylated CTCF6. Combined results of both analyses indicated that 12 tumours had LOH (10 hypermethylated CTCF6 and UPD 2 hypermethylated CTCF6 and hemizygous 11p15 deletion), 9 had LOI (hypermethylated CTCF6 and retention of heterozygosity (ROH)) and 22 had ROI (normally methylated CTCF6 and ROH). Of 21 tumours whose RNA was available, 9 and 12 tumours had heterozygous and homozygous *Apa*I/*Ava*II sites in exon 9 of *IGF2*, respectively. Of the nine heterozygous tumours, seven showed monoallelic expression of *IGF2*, indicating ROI, and two showed biallelic expression of *IGF2*, indicating LOI, and the results were consistent with those examined by COBRA and SNP array analyses (Table 1). From these findings, 11 tumours with normally methylated CTCF6, in which SNP array analysis was not performed, were classified as those with ROI. Thus, combined results of COBRA, SNP array and allelic expression analyses showed 12 tumours with LOH, 9 tumours with LOI and 33 tumours with ROI. In addition, one cell line (HuH6) had LOI, and the other (HepG2) had LOH (UPD) of *IGF2*.

The mean age was compared between any two of three groups of patients (i.e., LOH, LOI or ROI) by Student's *t*-test. There was no difference in the mean age between any two of the three groups of patients.

### Correlation between *IGF2* and *H19* mRNA levels and the *IGF2* status (LOH, LOI or ROI)

**Figure 2:**
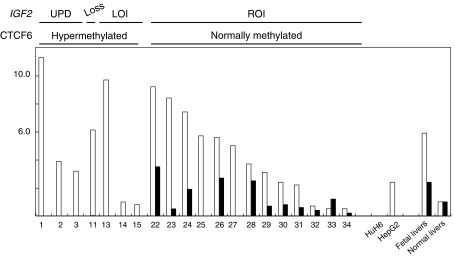
Results of quantitative real-time RT–PCR analysis of *IGF2* and *H19* mRNAs. Relative mRNA (*Y* axis) of total *IGF2* (open rectangles) and *H19* (closed rectangles) is plotted in 3 tumours with UPD, in 1 tumour with 11p15 loss, in 3 tumours with LOI, in 13 tumours with ROI, in 2 cell lines, in foetal liver total RNA and in adjacent normal liver tissues (a mean value of 3 samples). Tumours in each group are arranged in order by the levels of *IGF2* mRNA. Numbers below *X* axis indicate the tumour number shown in Table 1. *IGF2* status (UPD, loss of 11p15, LOI and ROI) and methylation status of CTCF6 at *H19* DMR (hypermethylated or normally methylated) are shown above the graph. Nine tumours (nos. 1–3, 11, 13–15, 25 and 27) and two cell lines expressed a minimal amount of *H19* mRNA, which was shown as zero in the graph. Similarly, HuH6 expressed a minimal amount of *IGF2* mRNA, which was shown as zero in the graph.

Quantitative real-time reverse transcription-PCR analysis showed that although 15 of 20 tumours had a higher level of *IGF2* mRNA than normal liver tissues, 15 of 20 tumours had a lower level of *H19* mRNA than normal liver tissues (Table 1 and Figure 2). All 3 tumours with UPD, 1 of 1 with 11p15 loss, 1 of 3 with LOI and 10 of 13 with ROI, expressed higher levels of *IGF2* mRNA than normal liver tissues. There was no significant difference in *IGF2* mRNA levels between 3 tumours with UPD or 7 tumours with *IGF2* alterations; that is UPD, 11p15 loss or LOI, and 13 tumours with ROI. In contrast, 7 tumours with *IGF2* alterations expressed very low levels of *H19* mRNA, whereas 11 of 13 tumours with ROI expressed a substantial amount of *H19* mRNA; 2 tumours (nos. 25 and 27) with ROI expressed very low levels of *H19* mRNA. *H19* mRNA levels were higher in 13 HB tumours with ROI than in 7 HB tumours with *IGF2* alterations (*P*<0.01 by Welch's *t*-test). Although HepG2 with UPD had a higher level of *IGF2* mRNA than normal liver tissues, HuH6 with LOI had a very low level of *IGF2* mRNA. *H19* mRNA levels were very low in both cell lines.

### Semiquantitative RT–PCR analysis of promoter-specific *IGF2* transcripts

**Figure 3:**
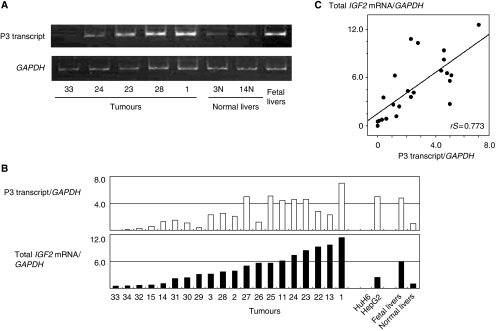
(**A**) Representative data of RT–PCR analysis of P3 transcripts. (**B**) Expression levels of P3 transcripts (upper lane) and total *IGF2* mRNA (lower lane) are plotted in 20 tumours, in 2 cell lines, in foetal liver tissues and normal liver tissues (a mean value of 3 samples). Tumours are arranged in order by total levels of *IGF2* mRNA. Numbers below *X* axis indicate the tumour number. (**C**) Correlation between levels of P3 transcript (*X* axis) and total *IGF2* mRNA (*Y* axis).

Because the *IGF2* gene has four kinds of promoters, promoter-specific *IGF2* transcripts were analysed to determine the usage of each promoter. Representative results of the P3 transcript are shown in Figure 3A. All 20 tumours showed undetectable or lower levels of P1 transcripts than 3 normal liver tissues. The levels of P2, P3 and P4 transcripts were higher in 13, 15 and 10 of the 20 tumours, respectively, than those of normal liver tissues. Polymerase chain reaction cycle numbers to obtain visible levels of PCR products were 40 for P2 transcripts, 30 for P3 transcripts and 35 for P4 transcripts, indicating that the amounts of P3 transcripts were high, those of P2 transcripts were low and those of P4 transcripts were intermediate. The Spearman correlation coefficient analysis showed that the expression levels of the P2, P3 and P4 transcripts correlated with the levels of total *IGF2* mRNA (P2, *rS*=0.730; P3, *rS*=0.773 and P4, *rS*=0.646) (Figure 3B and C; data for the P2 and P4 transcripts are not shown).

### The methylation status of *IGF2* promoters and its correlation with the levels of promoter-specific transcripts

**Figure 4:**
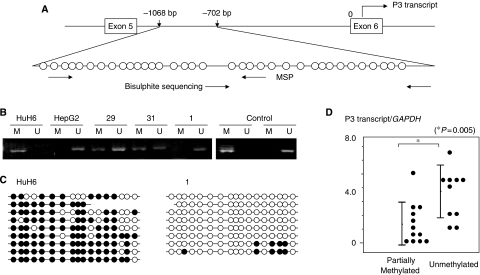
(**A**) Diagram of the *IGF2* P3 promoter region. Individual CpG dinucleotides located upstream of exon 6 (from −1068 to −702 bp) are represented by circles. Horizontal arrows indicate locations of PCR primers used for MSP and bisulphite sequencing. (**B**) Examples of the promoter methylation status using methylation-specific PCR. Polymerase chain reaction products of methylated or unmethylated P3 promoters from HB tumours are shown. Numbers above horizontal bars indicate the tumour number. M, methylated promoter; U, unmethylated promoter. (**C**) Bisulphite sequencing analysis of the methylation status of P3 promoter in HuH6 and one tumour (no. 1), which displayed complete methylation and complete unmethylation, respectively. Open and closed circles indicate unmethylated and methylated CpG dinucleotides, respectively. (**D**) Levels of P3 transcripts in tumours with partially methylated P3 promoter and tumours with unmethylated P3 promoter.

In the MSP analysis of each promoter, the P2 promoter region was partially methylated in 19 tumours and normal liver tissues and the P4 promoter region was unmethylated in all 20 tumours and normal liver tissues. Therefore, the methylation status of P2 or P4 promoter region was not correlated with the expression level of P2- or P4-specific transcripts. The P3 promoter region was partially methylated in 11 tumours, HuH6 and normal liver tissues and unmethylated in 9 tumours and HepG2 (Table 1, Figure 4A and B). The results of MSP analysis in one tumour (no. 1) and HuH6 were confirmed by bisulphite sequencing (Figure 4C). Nine tumours with the unmethylated P3 promoter had higher levels of P3 transcripts than 11 tumours with the partially methylated P3 promoter (*P*=0.005) (Figure 4D). The P3 promoter was unmethylated in 5 of 7 tumours with *IGF2* alterations; UPD, 11p15 loss or LOI, but in 4 of 13 tumours with ROI. Thus, the incidence of tumours with unmethylated P3 promoter tended to be higher in tumours with hypermethylated *H19* DMR than in tumours with normally methylated *H19* DMR (*P*=0.1).

### Semiquantitative RT–PCR analysis of *PLAG1* mRNA

**Figure 5:**
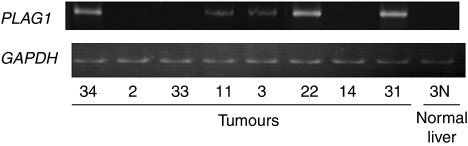
Representative data of RT–PCR analysis of *PLAG1* mRNA. Numbers below lanes indicate the tumour number.

*PLAG1* positively regulates *IGF2*, and its expression was detected in 12 tumours, foetal liver RNA and 2 cell lines, but not in 8 tumour and 3 normal liver tissues (Table 1 and Figure 5). The 12 tumours with *PLAG1* mRNA expression showed higher levels of P4-specific *IGF2* transcripts (*P*=0.01) and tended to show higher levels of P3-specific *IGF2* transcripts (*P*=0.051) than the 8 tumours without *PLAG1* expression. There was no significant difference in P2- or P1-specific transcript levels between tumours with and without *PLAG1* mRNA expression.

### Incidences of tumours with *CTNNB1* mutation between any two groups of tumours classified on the basis of the *IGF2* status

DNA was available for *CTNNB1* mutation analysis in 48 of 54 HB tumours. The results are described in Table 1. There were no differences in the incidences of *CTNNB1* mutation between 7 tumours with *IGF2*-LOI and 29 tumours with *IGF2*-ROI or 12 tumours with *IGF2*-LOH, and between 29 tumours with *IGF2*-ROI and 12 tumours with *IGF2*-LOH.

## Discussion

In this study, biallelic and monoallelic *IGF2* expressions correlated with hypermethylation and normal methylation of CTCF6, respectively, in two tumours with LOI and seven tumours with ROI (Table 1, Figure 1). In addition, the paternal origin of the duplicated *IGF2* loci was confirmed by the hypermethylated CTCF6 in 10 tumours with UPD. Furthermore, very low expression levels of *H19* mRNAs and substantial expression levels of *IGF2* mRNAs in HB tumours with UPD or LOI, and substantial expression levels of both *IGF2* and *H19* mRNA in HB tumours with ROI were found (Table 1 and Figure 2). Two (nos. 14 and 15) of three HB tumours with LOI expressed *IGF2* mRNA levels comparable to but not higher than those of *IGF2* mRNA in normal liver tissues. In addition, one cell line, HuH6, with LOI expressed minimal expression of *IGF2* mRNA, although Hartmann *et al* (2000) found the moderate expression in the same cell line. These findings may be explained by the speculation that such tumours expressed increased levels of *IGF2* mRNA at the critical time of tumorigenesis, but not at the time of surgical resection or after many passages of cell culture. From these findings, the hypothesis established for WT that the hypermethylation of maternal *H19* DMR causes LOI, and that LOI or duplication of paternal *IGF2* (UPD) results in overexpression of *IGF2*, may be also applied to HB.

Although the expression levels of *IGF2* mRNA were reported to be higher in WTs with UPD than in WTs with ROI in two series of WTs (Wang *et al*, 1996; Haruta *et al*, 2008), conflicting results were reported in *IGF2* mRNA levels between WTs with LOI and WTs with ROI (Wang *et al*, 1996; Ravenel *et al*, 2001). The present and earlier studies showed that all HB tumours with UPD and the majority of HB tumours with LOI or ROI expressed the higher levels of *IGF2* mRNA than normal liver tissues (Li *et al*, 1998b; Gray *et al*, 2000; Hartmann *et al*, 2000). This study also showed that P3 transcripts predominated in total *IGF2* mRNAs in HB tumours irrespective of the *IGF2* status (i.e., UPD, 11p15 loss, LOI or ROI); these findings were similar to those reported in foetal liver tissues showing elevated expression of *IGF2* mRNA with predominance of the P3 transcript (Li *et al*, 1998a). Thus, the high *IGF2* mRNA expression of many HB tumours with ROI may mimic the upregulation of *IGF2* expression in embryonic liver tissues, from which HB may arise.

In this study of 54 HB tumours, we found LOH in 12 (22.2%), LOI in 9 (16.7%) and ROI in 33 (61.1%). Hepatoblastoma tumours can be classified into those with LOH and those with ROH, and tumours with ROH can be further classified into those with LOI and those with ROI. For data comparison, the frequencies of LOH and LOI in the earlier and present series of HB tumours are shown in Tables 2 and 3, respectively (Davies, 1993; Montagna *et al*, 1994; Li *et al*, 1995; Rainier *et al*, 1995; Fukuzawa *et al*, 1999; Gray *et al*, 2000; Hartmann *et al*, 2000; Ross *et al*, 2000; Albrecht *et al*, 2004; Suzuki *et al*, 2008). Both frequencies of LOH and LOI were similar between the earlier and present series of HB tumours. When we compared the frequencies of LOH and LOI between HB and WT, the frequencies of LOH and LOI are lower in HB tumours than in WT tumours (Table 4). The present and earlier studies showed that levels of *IGF2* mRNA are higher in normal liver tissues than in normal kidney tissues, and in foetal liver tissues than in foetal kidney tissues, but showed similarly high levels in both WTs and HBs (part of the data not shown) (Hedborg *et al*, 1994; Haruta *et al*, 2008), indicating that embryonal kidney tissues might be more susceptible to IGF2 stimulation than embryonal liver tissues. These findings might be related to higher incidences of UPD or LOI in WT than in HB.

The *IGF2* gene has four promoter regions and each promoter can initiate transcription producing a distinct *IGF2* transcript with different 5′-untranslated regions with a common translated region in the 3′-side (Li *et al*, 1998a). The *IGF2* gene is transcriptionally regulated in a development-dependent and tissue-specific manner. In the foetal liver, promoters P2, P3, and P4 are active and expressed monoallelically; P3 is the most active promoter and P1 is inactive. However, in the adult liver, P1 becomes dominant and is biallelically expressed, and P2, P3 and P4 activities are decreased or lost (Li *et al*, 1998a). In foetal liver tissues, P3 promoter methylation is inversely correlated with the P3 transcript expression. The inverse correlation between P3 promoter methylation and P3 transcript expression was reported earlier in seven HB tumours (Li *et al*, 1998b). This study confirmed the upregulation of P2, P3 and P4 transcripts and downregulation of P1 transcript, and the inverse correlation between P3 promoter methylation and P3 transcript expression in the majority of 20 HB tumours. Although P2, P3 and P4 transcripts were all correlated to the total amount of *IGF2* mRNAs, the earlier and present studies showed that the P3 transcript was most abundant and seemed to play a major role in the tumorigenesis of HB (Li *et al*, 1998b). Increased *IGF2* expression with the predominant P3 transcript was reported earlier in WTs with LOI or ROI (Vu and Hoffman, 1999). This study also showed that HB tumours with hypermethylated *H19* DMR tended to have an unmethylated P3 promoter, indicating that the paternal P3 promoter or the maternal P3 promoter upstream of the aberrantly methylated *H19* DMR is likely to be unmethylated, probably because of stimulation of the enhancer signal. In contrast, the significance of unmethylation in the P3 promoter found in 4 (nos. 23, 24, 26 and 27) of 13 HB tumours with normally methylated *H19* DMR (ROI) remains unresolved.

*PLAG1* located in 8q11 encodes a developmentally regulated transcription factor, and positively regulates *IGF2*. The P3 promoter region of *IGF2* contains *PLAG1* consensus-binding sites, and PLAG1 transactivates the transcription from embryonic *IGF2* promoter P3 in HB cell lines, HuH6 and HepG2 (Zatkova *et al*, 2004). *PLAG1* mRNA was highly expressed in most HB tumours compared with normal liver tissues. In this study, HB tumours with *PLAG1* mRNA expression showed and tended to show higher levels of P4 and P3 transcripts, respectively. Thus, the correlation of *PLAG1* mRNA expression with increased levels of P3 transcripts reported by Zatkova *et al* (2004) may be confirmed; furthermore, the correlation of *PLAG1* mRNA expression with increased levels of P4 transcripts was also suggested.

WTs can be classified at least into two groups; one has intralobar nephrogenic rest that is associated with *WT1* abnormality and the other has perilobar nephrogenic rest associated with *IGF2*-LOI (Ravenel *et al*, 2001). *CTNNB1* mutation is frequently found in WTs with *WT1* abnormality, but rare in WTs without *WT1* abnormality (Maiti *et al*, 2000). These findings suggest that WTs with no *WT1* abnormality may include a substantial number of tumours with *IGF2*-LOI, and that *CTNNB1* mutation and *IGF2*-LOI may be mutually exclusive in WT and also in HB. However, there were no differences in the incidences of *CTNNB1* mutation between HBs with *IGF2*-LOI and those with *IGF2*-ROI, or those with *IGF2*-LOH. We have recently reported a paper describing the occurrence of duplication of paternal *IGF2* or *IGF2*-LOI in half of WTs with *WT1* abnormalities (Haruta *et al*, 2008). Of two WTs with *IGF2*-LOI and *WT1* abnormality reported in that paper, one had *CTNNB1* mutation and the other had not. These findings suggest that *CTNNB1* mutation and *IGF2*-LOI may not be mutually exclusive in either WT or HB.

The IGF signalling pathway is activated in various cancers, and monoclonal antibodies targeting IGF1R have been recently developed; IGF1R is a transmembrane tyrosine kinase receptor, and both IGF1 and IGF2 are ligands for IGF1R (Foulstone *et al*, 2005). Early clinical trials using anti-IGF1R monoclonal antibodies showed promising results in refractory Ewing's sarcomas and rhabdomyosarcomas (Ryan and Goss, 2008). Because 20–30% of HB tumours do not respond to the current chemotherapy consisting of cisplatin and adriamycin (Perilongo *et al*, 2000; Fuchs *et al*, 2002), and the great majority of HB tumours overexpresses *IGF2*, as shown in the present and earlier studies, HB may be the next target tumour for antibody therapy.

## Figures and Tables

**Table 1:** Genetic and epigenetic status of the *IGF2-H19* region in 54 hepatoblastoma tumours

**Patients number**	**Age^a^/sex**	**Chemo^b^**	**%methyl CTCF6^c^**	**11p15 SNP^d^**	***Apa*I site^e^**	***IGF2* RT-PCR**	***IGF2* status^f^**	***IGF2* mRNA**	**P1E^g^**	**P2M^h^**	**P2E**	**P3M**	**P3E**	**P4E**	***H19* mRNA**	***PLAG1* mRNA**	***CTNNB1* status^i^**
1	48/F	+	82.8	UPD	Homo	ND	UPD	11.3	0.6	MU	17.7	U	7	3.1	0	+	M
2	5/M	−	93.4	UPD	Homo	ND	UPD	3.9	0	MU	0	U	2.1	0.9	0	−	M
3	24/M	+	76.7	UPD	Homo	ND	UPD	3.2	0	MU	2.4	MU	2.3	1.2	0	+	M
4–10	5–96/M6, F1	+6, −1	72–90	UPD	ND	ND	UPD	ND	ND	ND	ND	ND	ND	ND	ND	ND	M4, N3
11	27/M	+	87.8	Loss chr II	Homo	ND	Loss	6.1	0	MU	2.3	U	4.4	1.3	0	+	M
12	24/M	+	81.9	Loss chr II	ND	ND	Loss	ND	ND	ND	ND	ND	ND	ND	ND	ND	M
13	12/F	+	91.4	ROH	Homo	ND	LOI (m)	9.7	0	U	1.1	U	2.3	0.6	0	−	ND
14	16/M	−	86.1	ROH	Homo	ND	LOI (m)	1	0	MU	0.3	U	1.3	0.9	0	−	ND
15	26/F	+	83.1	ROH	Hetero	LOI	LOI (m, p)	0.8	0	MU	0.4	MU	0.6	0.9	0	−	M
16	24/M	+	70.9	ROH	Hetero	LOI	LOI (m, p)	ND	ND	ND	ND	ND	ND	ND	ND	ND	M
17–21	12–84/M4, F1	+1, −3, UK1	71–91	ROH	ND	ND	LOI (m)	ND	ND	ND	ND	ND	ND	ND	ND	ND	M3,N2
22	12/F	+	52.5	ND	Homo	ND	ROI (m)	9.2	0.8	MU	8.4	MU	2.8	2.2	3.5	+	N
23	109/F	+	49.1	ND	Homo	ND	ROI (m)	8.4	0	MU	4.3	U	4.6	2.8	0.5	+	M
24	12/M	−	56.3	ND	Hetero	ROI	ROI (m, p)	7.4	0	MU	13.2	U	4.6	2.4	1.9	+	M
25	15/M	+	55.9	ROH	Hetero	ROI	ROI (m, p)	5.7	0	MU	6.5	MU	5.1	2.2	0	+	ND
26	6/M	UK	51.1	ND	Hetero	ROI	ROI (m, p)	5.6	0.1	MU	4.7	U	1.2	0.5	2.7	+	ND
27	10/M	+	62.1	ROH	Homo	ND	ROI (m)	5	0	MU	0.9	U	5	1.1	0	−	ND
28	29/F	+	61.5	ND	Homo	ND	ROI (m)	3.7	0	MU	1.5	MU	2.5	1.4	2.5	+	M
29	26/M	+	55.4	ND	Homo	ND	ROI (m)	3.1	0.2	MU	9.3	MU	0.4	1.2	0.7	−	M
30	18/M	+	48.7	ND	Hetero	ROI	ROI (m, p)	2.4	0.1	MU	1.2	MU	1.1	1	0.8	−	ND
31	13/M	+	55.3	ND	Hetero	ROI	ROI (m, p)	2.2	0	MU	1.1	MU	1.5	1	0.6	+	N
32	60/F	+	55.7	ND	Hetero	ROI	ROI (m, p)	0.7	0.2	MU	0.2	MU	0.3	0.9	0.4	+	N
33	29/M	+	56.4	ND	Homo	ND	ROI (m)	0.5	1	MU	0.1	MU	0	0.5	1.2	−	M
34	9/M	+	56.7	ND	Hetero	ROI	ROI (m, p)	0.5	0	MU	0	MU	0.1	0.2	0.2	+	M
35–54	4–156/M11, F9	+12, −7, UK1	41–65	ROH	ND	ND	ROI (m)	ND	ND	ND	ND	ND	ND	ND	ND	ND	M11, N9
Normal livers			52.8	ND	ND	ND	ROI (m)	1	1	MU	1	MU	1	1	1	−	
Fetal livers			ND		ND	ND	ND	5.9	0.2	ND	6.4	ND	4.8	1.9	2.4	ND	
HuH6			87.3	ROH	Hetero	LOI	LOI (m, p)	0	0	MU	0	MU	0	0.1	0	+	M
HepG2			89.5	UPD	Homo	ND	UPD (m)	2.4	0	U	1.5	U	5	2.8	0	+	M

F=female; M=male; M=methylated; ROH=retention of heterozygosity; ROI=retention of imprinting; U=unmethylated; ND=not done; UK=unknown.

All 20 tumours showed unmethylated promoter 4; UPD, uniparental disomy; loss chr 11, loss of chromosome 11or 11p15; LOI, loss of imprinting.

a Age in months.

b Chemo, chemotherapy before surgery; +6, −1 indicates that six and one tumours were treated and untreated, respectively, with chemotherapy before surgery.

c %methyl CTCF6 indicates % methylated CTCF6 allele.

d Results of SNP array.

e Homo, homozygosity at *Apa*I/*Ava*II site; hetero, heterozygosity.

f Results of SNP array analysis, methylation analysis of CTCF6 (m) and *Apa*I/*Ava*II polymorphism site analysis (p).

g P1E, promoter 1-specific transcript.

h P2M, the methylation status of promoter 2.

i *CTNNB1* status: M, mutated; N, normal.

**Table 2:** Incidences of LOH of *IGF2* in previous and present series of hepatoblastoma and Wilms' tumours

**References**	**Total number**	**LOH of *IGF2*^a^**	**No-LOH of *IGF2***	**%**
*Hepatoblastoma*				
Montagna *et al* (1994)	13	3	10	23.1
Fukuzawa *et al* (1999)	7	2	5	28.6
Gray *et al* (2000)	10	2	8	20.0
Hartmann *et al* (2000)	24	6	18	25.0
Albrecht *et al* (2004)	56	13	43	23.2
Suzuki *et al* (2008)	17	4	13	23.5
Total number	127	30	97	23.6
Present study	54	12	42	22.2
				
*Wilms' tumour*				
Grundy *et al* (1996)	260	93	167	35.8
Yuan *et al* (2005)	62	26	36	41.9

a Tumours with LOH of 11p15, but no informative *IGF2* locus are included.

**Table 3:** Incidences of LOI of *IGF2* in previous and present series of hepatoblastoma and Wilms' tumours

**References**	**Total number^a^**	**LOI of *IGF2***	**ROI of *IGF2***	**%**
*Hepatoblastoma*				
Davies (1993)	3	0	3	0
Montagna *et al* (1994)	5	1	4	20.0
Rainier *et al* (1995)	5	1	4	20.0
Li *et al* (1995)	3	1	2	33.3
Fukuzawa *et al* (1999)	4	1	3	25.0
Ross *et al* (2000)	13	3	10	23.1
Hartmann *et al* (2000)	5	3	2	60.0
Total number	38	10	28	26.3
Present study	42	9	33	21.4
				
*Wilms' tumour*				
Ravenel *et al* (2001)	36	15	21	41.7
Yuan *et al* (2005)	29	22	7	75.9

a Tumours with LOH of *IGF2* were excluded.

**Table 4:** Incidences of LOH, LOI and ROI of *IGF2* in hepatoblastoma and Wilms' tumours

**References**	**Total number**	**LOH of *IGF2***	**LOI of *IGF2***	**ROI of *IGF2***
*Hepatoblastoma*				
Present study	54	12 (22.2%)	9 (16.7%)	33 (61.1%)
				
*Wilms' tumour*				
Fukuzawa *et al* (2004)	41	17 (41.5%)	13 (31.7%)	11 (26.8%)
Yuan *et al* (2005)	58	29 (50.0%)	22 (37.9%)	7 (12.1%)
